# The extent of mangrove change and potential for recovery following severe Tropical Cyclone Yasi, Hinchinbrook Island, Queensland, Australia

**DOI:** 10.1002/ece3.4485

**Published:** 2018-10-16

**Authors:** Emma Asbridge, Richard Lucas, Kerrylee Rogers, Arnon Accad

**Affiliations:** ^1^ Centre for Ecosystem Sciences, Biological and Environmental Sciences the University of New South Wales Kensington New South Wales Australia; ^2^ School of Earth and Environmental Science University of Wollongong Wollongong New South Wales Australia; ^3^ Department of Science, Information Technology and Innovation Queensland Herbarium Brisbane Botanic Gardens Mt Coot‐tha Toowong, Brisbane Queensland Australia

**Keywords:** biodiversity, climate change, cyclone, mangroves, remote sensing

## Abstract

Cyclones are significant drivers of change within mangrove ecosystems with the extent of initial damage determined by storm severity, location and distribution (exposure), and influenced by species composition and structure (e.g., height). The long‐term recovery of mangroves is often dependent upon hydrological regimes, as well as the frequency of storm events. On February 3, 2011, Tropical Cyclone Yasi (Category 5) made landfall on the coast of north Queensland Australia with its path crossing the extensive mangroves within and surrounding Hinchinbrook Island National Park. Based on a combination of Landsat‐derived foliage projective cover (FPC), Queensland Globe aerial imagery, and RapidEye imagery, 16% of the 13,795 ha of mangroves experienced severe windthrow during the storm. The greatest damage from the cyclone was inflicted on mangrove forests dominated primarily by *Rhizophora stylosa*, whose large prop roots were unable to support them as wind speeds exceeded 280 km/hr. Classification of 2016 RapidEye data indicated that many areas of damage had experienced no or very limited recovery in the period following the cyclone, with this confirmed by a rapid decline in Landsat‐derived FPC (from levels > 90% from 1986 to just prior to the cyclone to < 20% postcyclone) and no noticeable increase in subsequent years. Advanced Land Observing Satellite (ALOS‐1) Phased Arrayed L‐band Synthetic Aperture Radar (SAR) L‐band HH backscatter also increased initially and rapidly to 5 ± 2 dB (2007–2011) due to the increase in woody debris but then decreased subsequently to −20 ± 2 dB (postcyclone), as this decomposed or was removed. The lack of recovery in affected areas was attributed to the inability of mangrove species, particularly *R. stylosa,* to resprout from remaining plant material and persistent inundation due to a decrease in sediment elevation thereby preventing propagule establishment. This study indicates that increases in storm intensity predicted with changes in global climate may lead to a reduction in the area, diversity, and abundance of mangroves surrounding Hinchinbrook Island.

## 1 INTRODUCTION

Mangrove forests are distributed in the intertidal zone (usually from mean sea level to highest spring tide; Alongi, 2009) along subtropical and tropical coastlines, between approximately 30°N and 30°S latitude (Giri et al., 2011). Using Earth Observation (EO) satellite data in 2000 mangroves were identified in 118 countries and occupied a global area of 137,760 km^2^ (0.7% of global tropical forests), with 75% located in 15 countries. However, across their global range, mangroves are experiencing degradation and loss partly due to anthropogenic activities (i.e., land clearance for agriculture, aquaculture, and urban expansion) and natural causes (i.e., changes in climate and environmental processes; Thomas et al., 2017; Over 3.6 million ha (20%) have been lost globally since 1980 (FAO, 2007), with several regions experiencing major destruction (e.g., South India, Puerto Rico, and Singapore, with losses of 6%, 89%, and 74%, respectively (Mastaller, 1996)). The forests within Southeast Asia are of particular concern as despite having the greatest diversity (Spalding, Kainuma, & Collins, 2010) and over one‐third of the total global extent (Giri et al., 2011), rapid and extensive deforestation was responsible for the loss of more than one‐third of the forests between 1980 and 1990 (Valiela, Bowen, & York, 2001). Between 2000 and 2012, the forests were lost at an average rate of 0.18% per annum primarily due to clearance for aquaculture and rice and oil palm agriculture (Richards & Friess, 2016). Global loss has led to 11 of the 70 mangrove species being listed on the International Union for the Conservation of Nature (IUCN) Red List of Threatened Species (in June 2010) and a further six classed as “vulnerable” (FAO, 2007, Polidoro et al., 2010).

Mangrove loss and gain in extent and condition are also associated with natural events (e.g., intense storms, tsunamis) and processes (e.g., changing sea levels, ocean circulations climate; (Asbridge, Lucas, Accad, & Dowling, 2015; Asbridge & Lucas, 2016; Gilman, Ellison, Duke, & Field, 2008). Increasingly, natural processes are being exacerbated by drivers associated with climatic changes, including those linked with human activities (McKee, Rogers, & Saintilan, 2012). The impacts of climate change on mangroves are difficult to quantify where they have been lost or degraded by human activities, but are more evident in areas that have remained relatively undisturbed as a consequence of isolation or protection (Thomas et al., 2014).

In different regions of the world, intense storms are a strong driving force for change in mangrove ecosystems (Lugo, 2000). Cyclonic activity can alter forest structure and function and effect biodiversity, nutrient cycling, and sediment dynamics (Baldwin, Egnotovich, Ford, & Platt, 2001; Herbert, Fownes, & Vitousek, 1999). Contributing factors include high winds, fluctuations in sea level (e.g., storm surges), strong waves, and changes in hydrology and sediment distributions through erosion and accretion (Cahoon & Hensel, 2002). The vulnerability of mangroves to loss or damage from storms is influenced by their location and particularly exposure (Baldwin et al., 2001; Platt, Doren, & Armentano, 2000), with those occurring along estuaries and saltwater lagoons or protected by mountain ranges being less exposed to the full force of storms. However, it is still unclear how the physical and biological processes interact to impact mangrove mortality and the severity of degradation in relation to storm dynamics and intensity (Doyle, Smith, & Robblee, 1995). The recovery of mangroves from storm events depends upon the ability of different mangroves species to resprout from existing plant material or propagules to reach the affected sites.

**Figure 1:**
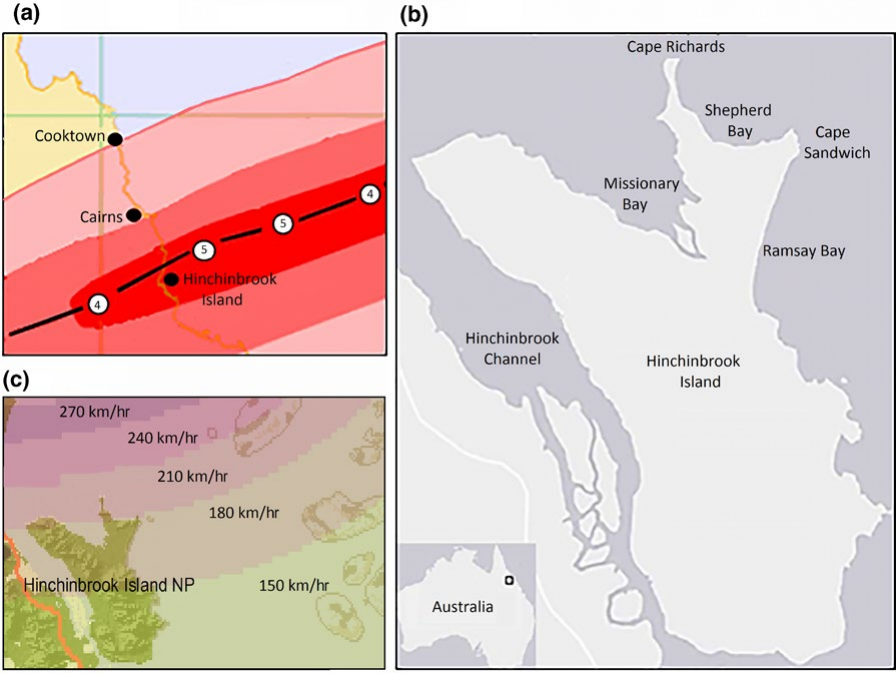
(a) Track and intensity of Cyclone Yasi (BOM, 2011), (b) location of Hinchinbrook Island, and (c) average wind speed zones from Cyclone Yasi (NSPR, 2011)

**Figure 2:**
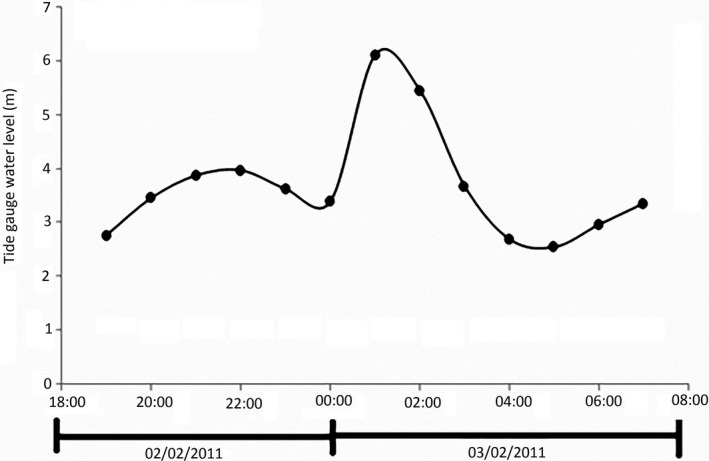
The water level measured at Cardwell tide gauge indicating a peak when Cyclone Yasi crossed the coastline (Queensland Government, 2015)

On February 3, 2011, Tropical Cyclone Yasi developed into a Category 5 storm as measured on the Australian Bureau of Meteorology scale (Figure 1b) (Category 4 on the Saffir–Simpson Scale) as it crossed the north Queensland coastline of Australia near Mission Beach between midnight and 1 a.m. Landfall was 40 km north of Hinchinbrook Island (Figure 1a), which supports the largest contiguous area of mangroves in Australia. The 500‐km‐diameter cyclone (with an eye of 30 km) generated wind gusts up to 285 km/hr, and maximum wind speeds were estimated at 215 km/hr (10 min average). Average wind strength zones are shown in Figure 1c. The resultant storm surge caused a temporary sea‐level rise of 7 m (Beeden et al., 2015), and 12 m offshore waves and 6 m near‐shore waves (Haigh et al., 2014). The cyclone crossed land during high tide, which further exacerbated the already enhanced water levels measured at the Cardwell tide gauge. The water level increased from 2.773 m at 7 p.m. (February 2) to 6.113 m at 1 a.m. (February 3, 2011) (Figure 2). Cyclone Yasi and Cyclone Larry (Category 4, March 2006) are considered the most severe cyclones to cross the coast between Cairns and Cardwell since an unnamed cyclone in 1918 (Category 5) (Turton & Stork, 2008). Turton (2012) estimates the cyclone return interval at once in 70 years for the tropical region of northern Queensland. Figure 1. a) Track and intensity of Cyclone Yasi (Bureau of Meteorology, 2011), b) location of Hinchinbrook Island, and c) average wind speed zones from Cyclone Yasi (NSPR, 2011).

The cyclone inflicted considerable damage on the mangroves surrounding Hinchinbrook Island. The Queensland Government reported that 17.2% of the precyclone extent was affected by the cyclone. Aerial surveys north of (but not including) mangroves around Hinchinbrook Island in 2011 and 2013 were undertaken to visually assess (using approximately 500 photographs) the extent of damage to major vegetation communities and the extent of recovery. The study concluded that vast areas of mangrove had experienced dieback with this potentially attributed to the inability of some mangroves species to adapt to the variation in salinity associated with changes in sediment dynamics, hydrology, and a storm surge. Previous studies confirm that although many species are tolerant to changes in salinity, a number of species experience reduced photosynthesis and growth (Lin & Sternberg, 1993). One of the proposed solutions for recovery at Hinchinbrook Island is to “let nature take its course” (Holloway, 2013).

Within Australia, there has been increasing concern regarding the impacts of climate change on the long‐term health of mangroves and the impacts on biodiversity as well as society and economics. This issue has gained greater prominence following the recent dieback event (Duke et al., 2017), which affected over 10,000 ha of mangroves in northern Australia. This was linked to a reduction in sea level in accordance with a strong El Niño Southern Oscillation (ENSO) event (Lovelock, 2017, Saintilan, Rogers, Kelleway, Ens, & Sloane, 2018; Lucas et al., 2018). Climate change projections indicate an increase in the intensity of tropical cyclones (IPCC, 2013), with Cyclone Yasi potentially being an example as it was the largest intensity storm to cross the north Queensland coast since 1918 (Bureau of Meteorology, 2011). The concern is that changes in cyclone regimes in northern Australia might have a long‐term impact on the viability of mangrove communities and compromise their ability to support ecosystem services. For this reason, this study aimed to:

1. Quantify the loss and degradation of mangroves surrounding Hinchinbrook Island during and following Tropical Cyclone Yasi through time‐series comparison of very high‐resolution aerial and RapidEye imagery and temporal sequences of space‐borne optical and Japanese Aerospace Exploration Agency (JAXA) L‐band Synthetic Aperture Radar (SAR) data.
2. Establish the extent to which mangroves had recovered from the cyclone.
3. Suggest a number of hypotheses to explain the patterns of mangrove mortality and recovery and provide an insight into the long‐term recovery of mangroves.

Short‐ and long‐term impacts of cyclone activity and recovery trajectories are discussed below with both global and local examples. The location, land use, climate, ocean circulation, tidal regime, and biodiversity of Hinchinbrook Island are presented, and the available data and methods of data analysis are described. The results present a time series of FPC and HH and HV backscatter for the designated land cover classes and provide maps of mangrove loss. The reasons for change in mangrove structure and health and the patterns of destruction and recovery are discussed with reference to global and future implications.

## 2 BACKGROUND

**Figure 3:**
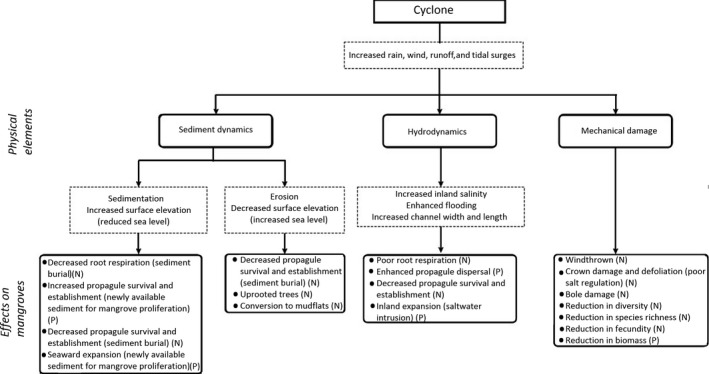
The physical changes following cyclonic activity and the resultant positive (P) and negative (N) implications for mangroves

Tropical storms (cyclones, typhoons and hurricanes) are common along the World's tropical and subtropical coastlines and can exert considerable damage to mangroves, which may be wind and/or water driven. The following sections provide an overview of the impacts of these storms over varying time frames and focus on mechanical damage, sediment erosion and accretion, inundation and salinity changes. An overview of the physical changes in the environment together with the positive and negative implications for mangroves forests is presented in Figure 3. Although this investigation does not directly measure the changes to sediment and hydrological dynamics, this figure provides a clear summary of the processes affected by direct and indirect cyclonic activity. It is important to understand the array of positive and negative implications, in order to comprehend the complexity of the short‐ and long‐term recovery trajectories.

### 2.1 Mechanical damage

In the short term, mangroves experience direct destruction from wind action, including windthrow, crown damage (defoliation and snapping of small to large branches), bole damage, and mortality (Kjerfve, 1990; Stocker, 1976). In the long term, changes in the distribution, species composition, and growth can occur as a consequence of changes in sediment dynamics (erosion, accretion) and hydrology (inundation, drainage (Jimenez, Lugo, & Cintron, 1985)); these secondary effects may only become apparent months after the storms and may persist for many years (Gilman et al., 2008; Smith, Robblee, Wanless, & Doyle, 1994).

### 2.2 Sediment redistribution

#### 2.2.1 Erosion

During the storm event, strong winds, currents, and waves can lead to significant erosion of the substrate material (Swiadek, 1997) and undercutting of mangroves. As an example, significant erosion occurred in Bowling Green Bay south of Townsville, north Queensland, and along the eastern region of the Burdekin Delta following Cyclone Althea in December 1971 when water overtopped ridges and banks, with many mangroves uprooted or sand‐blasted by the wind. Although the majority of mangroves were able to recover, the complete burial of many pneumatophores resulted in mass mortality in the following year (Hopley, 1974). Similarly, following major flooding in January 2011, 92% of the mangroves along 76 km of the Brisbane River experienced mortality as a result of inundation and erosion (Asbridge et al., 2015; Dowling, 2012). Following Hurricane Andrew in Florida in 1992, erosion of sediments (by an average of 2–3 cm (Cahoon et al., 2003)) led to the uprooting of trees which further exposed previously protected land behind the forest to wind and wave action (Doyle et al., 1995; Swiadek, 1997). Collapse of sediments can continue until affected areas are stabilized by roots from newly established propagules or surviving mangroves.

#### 2.2.2 Accretion

During and following large storms, substantive movement of eroded sediments is typical and where the subsequent deposition is excessive, mangroves may suffer because of the reduced ability to respire from roots and oxygen deficit stress particularly where this is in excess of >10 cm/year above normal rates. The rate and extent of mortality depend upon the degree of burial, with species with prop roots (e.g., *Rhizophora* and *Bruguiera*) being more tolerant compared to those with pneumatophores (e.g., *Avicennia* spp.; (Ellison, 1999; Paling, Kobryn, & Humphreys, 2008)). Once the roots have died, the sediment and decomposing roots can become anoxic, resulting in a decrease in the redox potential and an increase in the concentration of sulfides (Mendelssohn, Kleiss, & Wakeley, 1995). Without the supportive root systems, sediment elevations may reduce through decomposition of organic matter but the amount will vary within the mangrove system. For example, basin mangroves often experience sediment collapse over a longer period of time compared to fringe mangroves because of differences in sediment structure and the different susceptibilities of mangroves to dieback. Where the growth rates and densities of roots (particularly fine) are high (as in the case of *Rhizophora* species that often dominate fringe mangroves) and rates of root decomposition are low, sediment strengths tend to be greater (Cahoon et al., 2003; Middleton & Mckee, 2001). Hence, forests dominated by species such as *Rhizophora* may be less vulnerable to collapse and recovery may be greater compared to other species occurring in other elevation zones. Sedimentation following cyclones can also alter flushing regimes, as a consequence of changes in topography. Areas of standing water may remain where sediment obstructs flows, with this further reducing oxygen concentration and redox potential (Bardsley, Davie, & Woodroffe, 1985; Mendelssohn et al., 1995).

### 2.3 Inundation

During the early phases of inundation, the influx of water can remove litter layers and propagules which reduces recruitment, productivity, and in situ carbon cycling (Forbes & Cyrus, 1992). Where inundation occurs for long periods of time, the health and productivity of mangroves can be affected. As examples, a cyclone struck the Kosi Estuary in South Africa in January 1966, which resulted in a 6 m rise in water level and when Cyclone Domoina made landfall at Natal, South Africa, in January 1984, large areas of the two main river channels were eroded with the rivers retreating by 100 m and widening by 300 m in some locations, with the depth increasing by 10–14 m. The increased amount of water led to significant mortality of mangroves (Breen & Hill, 1969; Forbes & Cyrus, 1992) and, in the latter case, the magnitude related directly to the period of submersion and the height of mangroves. Many of the smallest saplings (<1 m) succumbed after several days to weeks whereas those up to 3.5 m in height remained alive for longer, although suffered mortality when inundation extended to 4 months (Steinke & Ward, 1989). This mortality occurs in part because gas exchange through the aerenchyma of pneumatophores is prevented. The extent and magnitude of mortality also vary as species have different tolerances to prolonged periods of submersion. For example, mortality may be higher for species with pneumatophores (e.g., *Avicennia*) because the aerenchyma are unable to facilitate gaseous exchange. In this regard, cyclones may reduce or alter species richness, diversity, complexity, and overall resilience of the forests (Baldwin, Platt, Gathen, Lessmann, & Rauch, 1995; Rey, Crossman, & Kain, 1990), and, in some cases, mangroves can disappear altogether.

### 2.4 Salinity changes

Mangroves are halophytes, thriving in saline conditions whereby salinity plays a significant role in regulating the growth and distribution of the forests (Waisel, 1972). Inundation resulting from storm surges and changed hydrological dynamics following cyclonic activity is associated with increased salinity and saltwater intrusion. High salinity can limit growth rates, productivity, propagule production, fecundity, seedling survival, and decrease hydraulic conductivity, leaf water potential, stomatal conductance, and photosynthesis (Ball, 1998; Lovelock, Ball, Feller, Engelbrecht, & Ling Ewe, 2006; Lovelock et al., 2011; Morrisey et al., 2010). Saline tolerance varies among species due to their morphological and physiological adaptations. In this way, the abrupt salinity change and continuous inundation may render mangroves unable to make physiological adjustments, potentially resulting in reduced forest diversity, structure, and overall resilience.

### 2.5 Recovery of mangroves

Mangrove recovery is often facilitated by growth from reserve or secondary meristematic tissues, sprouting from trunks or branches, or establishment from propagules. Such growth is variable and depends on the extent and severity of damage, water flows during and following the cyclone (removing or depositing propagules), and the species type (Imbert, Rousseau, & Labbe, 1998; Smith et al., 1994). Recovery also depends upon the rate of peat collapse which, when combined with poor propagule production, prevents rapid colonization and leads to sediment instability, reductions in elevation, and subsequent tidal inundation, factors that all limit further propagule establishment (Lugo & Patterson‐Zucca, 1977).

## 3 STUDY AREA

Hinchinbrook Island is one of Australia's largest national parks (39,350 ha), located in the Coral Sea 8 km southeast of Cardwell, Queensland (18.33°S, 146.22°E) (Figure 1b). The Island is only accessible via boat and is uninhabited with the exception of a small tourist resort at Cape Richards. The predominant land use is tourism, principally marine‐based activities such as sailing, scuba diving, fishing, and swimming.

Hinchinbrook Island is mild subtropical with “wet” and “dry” seasons. The high rainfall on the island is attributed to moist southeasterly winds rising over the central mountains. This rise in air results in adiabatic cooling, condensation, and precipitation (orographic rainfall) (Pye & Mazzullo, 1994; Van Riper, Kyle, Sutton, Barnes, & Sherrouse, 2012).

Queensland experiences, on average, four to five cyclones per year, coinciding with ENSO, yet many of the systems remain offshore without crossing the coastline. Semidiurnal tides in the Hinchinbrook region reach a maximum range of 3.5 m (Alongi, 1994; Pye & Mazzullo, 1994). During the dry season from March to November, the prevailing trade winds (typically >15 knots) travel over a comparatively short fetch, in a southeasterly direction creating short wind waves (rarely >1 m) and a southerly tidal flow (Pye & Rhodes, 1985). By contrast, large storm waves are produced in the wet season as a result of cyclonic activity (Belperio, 1978; Pye & Rhodes, 1985). The southeasterly trade winds generate a longshore current in a northerly direction along the inshore of the island.

Hinchinbrook Island is one of the most biologically diverse and species‐rich continental islands in the Great Barrier Reef with 54 ecosystems (Stanton & Godwin, 1989) (46 are declared as being of concern or endangered, with the four remaining not found in any other protected regions in Queensland; (Van Riper et al., 2012)). There are vast and diverse mangrove stands, with 31 species identified that form structurally diverse communities ranging from stunted/dwarf regions to mature forests with canopy heights >40 m (Bunt, Williams, & Clay, 1982; Ellison, 2000). Dominant species include *Rhizophora apiculata, R. stylosa, Rhizophora lamarckl*i, *Ceriops australis, Ceriops tagal,* and *Bruguiera gymnorhiza* (Boto & Bunt, 1981; Clough, 1998). The two largest regions of mangrove forest occur at Missionary Bay (approximately 20 km^2^) and within the Hinchinbrook Channel (approximately 164 km^2^), which together denote one of the largest neighboring mangrove forests in Australia (Clough, 1998). In 1997, the mangroves on Hinchinbrook Channel were, on average, >50 years old and approximately 10 m in height (Duke, 1997). From 1943 to 1991, there was no significant change in the total area of mangrove or saltmarsh at the island in the Hinchinbrook Channel. However, there was a substantial net change in the proportions of intertidal vegetation, as 78% of the saltmarsh (and some small areas of short mangroves) were replaced by tall mangrove forests, with this change attributed to variations in rainfall (Ebert, 1995; cited in Duke, 1995). Mangroves also proliferate in the sheltered sand dunes at Ramsay Bay on unconsolidated sediments (colluvial and alluvial) and in between Deluge and Gayundah Inlets (Queensland Parks and Wildlife Service, 2016).

The distribution of mangroves on Hinchinbrook Island is partly controlled by exposure, with the seaward (exposed) side of the island devoid of mangroves, except for a sheltered area behind a sand barrier. In contrast, the western side is protected and has extensive forests (Galloway, 1982). In addition, tidal action also influences distribution via the dispersal and establishment of propagules. For instance, at the northern end of the island the mangroves extend 5 km along the sheltered side of a tombolo and are distributed adjacent to tidal channels. Within the sheltered Hinchinbrook Channel, the forests follow the complex network of tidal channels and exhibit explicit zonation (Galloway, 1982). The tidal dynamics surrounding the island are asymmetrical partly due to the mangroves forests (Wolanski, Jones, & Bunt, 1980). At the southern end of the island, the flood tidal current flows in a northerly direction, whereas at the northern end of the island the current is southerly. This allows the tidal channel to remain open and deep through self‐scouring (Galloway, 1982).

The forests are predominantly influenced by saline water, with freshwater influxes only experienced during periods of intense/persistent rainfall and/or low tide (Lear & Turner, 1977). However, the island within Hinchinbrook Channel is influenced by river discharge from both the mainland and runoff from Hinchinbrook Island.

## 4 METHODS

### 4.1 Available data

#### 4.1.1 Landsat foliage projective cover

To determine changes in foliage cover following Cyclone Yasi, an annual time series of Landsat‐derived foliage projective cover (FPC) from 1987 to 2016 was obtained from the Queensland Department of Science, Information Technology and Innovation (DSITI). FPC is defined as the proportion of a pixel containing the vertical projection of vegetation (Armston, Denham, Danaher, Scarth, & Moffiet, 2009). FPC mapping only uses dry season (May to November) imagery in an automated classification at 25 m resolution. The FPC for mature mangroves is usually > 80% because of the high density of trees with a full canopy and a threshold of 30% FPC (equating to 50% canopy cover) can consistently delineate the majority of mangroves from other vegetation and mudflats (Asbridge & Lucas, 2016).

#### 4.1.2 ALOS

Advanced Land Observing Satellite (ALOS) Phased Arrayed L‐band SAR (PALSAR) Fine Beam Single (FBS; HH polarization), Dual (FBD; HH and HV polarization), and Polarimetric (PS; HH, VV, HV) data were acquired over Hinchinbrook Island prior to and following Cyclone Yasi by the Japanese Space Exploration Agency (JAXA). These were made available through the Japanese Space Exploration Agency (JAXA's) Kyoto and Carbon (K&C) Initiative. The ALOS‐PALSAR data were converted to Gamma0 using coefficients provided by JAXA (Shimada, Isoguchi, Tadono, & Isono, 2009).

L‐band microwaves penetrate through foliage and interact predominantly with the woody parts of the mangrove (i.e., the trunk and branches). Waves that are transmitted horizontally and return to the sensor in a vertical orientation (HV) indicate volume scattering within the branches of the canopy. Horizontally transmitted waves returning in a horizontal orientation (HH) have typically experienced double‐bounce interaction, between the trunks and the sediment. ALOS‐PALSAR data are influenced by vegetation water content, including that on that associated with rainfall (Lucas et al., 2010).

#### 4.1.3 Digital photography and RapidEye

RapidEye data acquired in 2014 were made available through the Planetlabs Ambassador Program. The recent acquisition of the data, which has a resolution of 6.5 m provided an opportunity to map the extent of damage to mangroves following Cyclone Yasi. However, high tide prevented the sole use of RapidEye data for land cover classification, particularly as nonvegetated areas such as mudflats were not visible. The RapidEye data were therefore used in combination with digital aerial photography provided by the Queensland Globe as open access data within Google Earth. The imagery was provided by the Department of Natural Resources and Mines.

#### 4.1.4 Ancillary data

Daily rainfall data were obtained over the period of the ALOS‐PALSAR acquisitions at Cardwell Range (146.18°E, 18.55°S) to establish whether variations in L‐band SAR backscatter were attributed to changes in rainfall rather than actual vegetation cover. To define the extent of mangroves prior to the cyclone, Regional Ecosystem maps were obtained via the Queensland Herbarium. In addition, a map of Hinchinbrook National Park was also acquired from the Department of National Parks, Sport and Racing.

### 4.2 Data processing and analysis

The regional ecosystem map was used to define the wetland region surrounding Hinchinbrook Island, which included mangroves and mudflats. This region was then classified into four broad land cover classes (surviving mangroves, dead mangroves, other vegetation (i.e., rainforest), and nonvegetated mudflats) by applying a maximum‐likelihood classification to RapidEye (2015) data. Randomly sampled points were located and allocated to the four land cover classes by referencing Landsat FPC (2009 and 2015) and Queensland Globe (2016) imagery. These were used to train the classifier and subsequently determine the accuracy of the classification.

**Figure 4:**
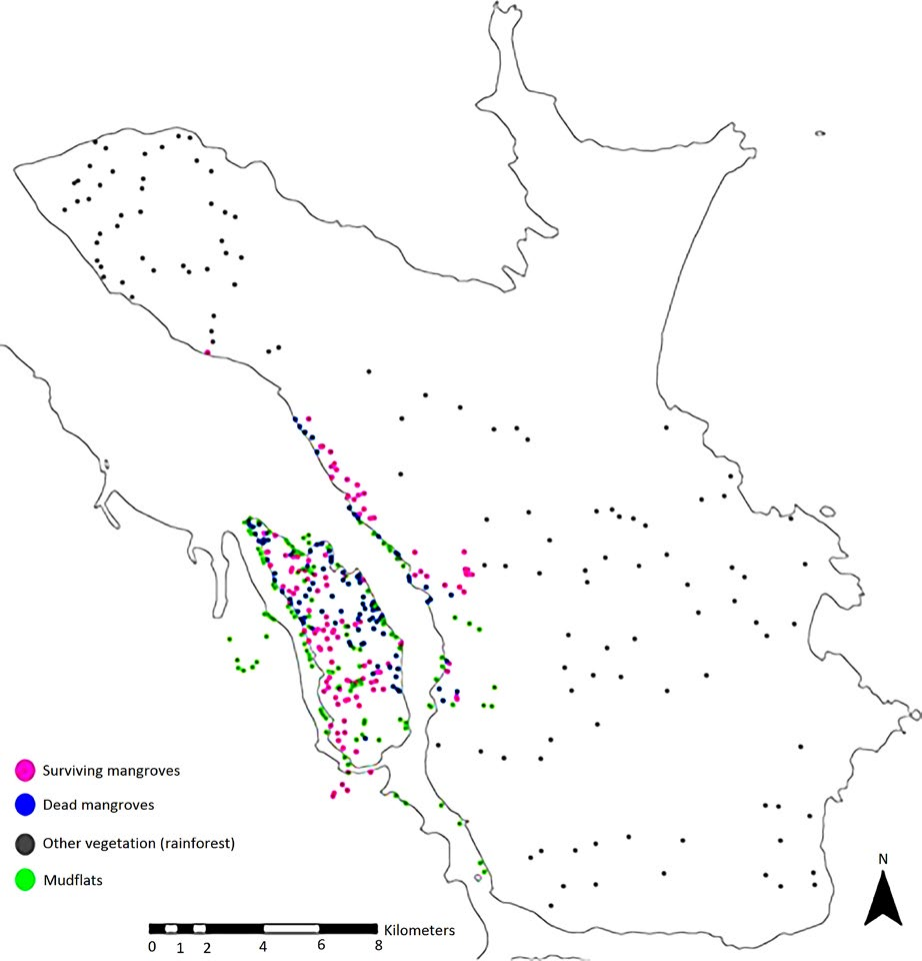
Location of regions of interest representing (a) surviving and (b) dead mangroves, (c) other vegetation (rainforest) and (d) mudflats

For each class, 120 regions of interest (ROIs; typically <10 ha in size) were delineated and statistics for FPC and L‐band HH and HV data were extracted subsequently (Figure 4). Training areas were established for areas of dead mangroves/clearings (mudflats) and healthy mangroves prior to (2009) and following (2015) Cyclone Yasi. By using a combination of different types of satellite observation data, mangroves could be delineated as dead or alive. This is useful, as FPC data can only indicate the degree of defoliation. However, when combined with high‐resolution RapidEye data and Queensland Globe imagery areas with dead trees (woody debris) were able to be defined.

Using the change maps, the areas of mangrove loss were calculated, together with the percentage change for Hinchinbrook Island (National Park zone), the island within Hinchinbrook Channel and the Queensland coastline. An accuracy assessment was conducted using randomly sampled points for surviving and dead mangroves. The percentage change in the FPC prior to (2009) and following (2015) the cyclone was mapped using the following equation: ((FPC 2009 ‐ FPC 2015)/FPC 2009)*100. The regions of surviving and dead mangroves, created from the maximum‐likelihood classification, were overlain onto the FPC change map to identify the presence or absence of recovery.

## 5 RESULTS

### 5.1 Extent of damage to mangroves, post‐Cyclone Yasi

**Figure 5:**
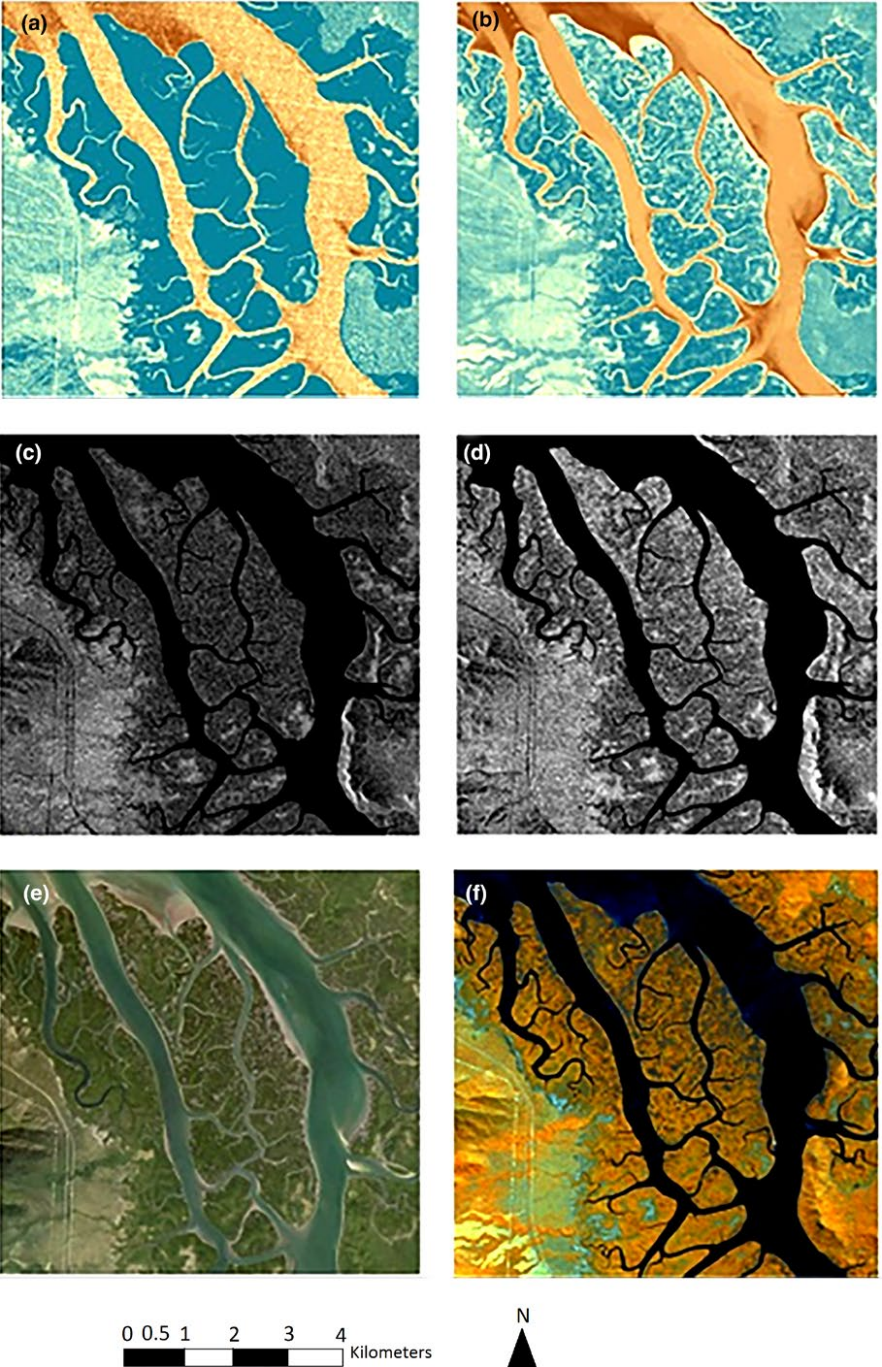
The extent of mangrove damage prior to and following cyclone Yasi. (a) FPC: June 1, 2010, (b) FPC: September 13, 2013, (c) ALOS: February 2, 2010, (d) ALOS: November 12, 2014, (e) Queensland Globe: March 8, 2015, and (f) RapidEye: May 3, 2014

The extent of damage to mangroves at the island within the Hinchinbrook Channel is evident in Figure 5. The defoliation was identified by comparing FPC images before (Figure 5a) and after (Figure 5b) the cyclone. The reduction in FPC (i.e., defoliation) is represented by a paler blue coloration with cream/yellow patches). The damage to the woody components of the trees (i.e., branches, and trunks) is seen by comparing the ALOS images before (Figure 5c) and after (Figure 5d) the cyclone. The brighter white region in Figure 5d indicates fallen trees. The Queensland Globe (Figure 5e) and RapidEye (Figure 5f) imagery were only available following the cyclone. The patches of dead/damaged mangroves are visible as dark areas in among the healthy forest represented as green in Figure 5e and yellow in Figure 5f.

### 5.2 Changes in Landsat FPC

**Figure 6:**
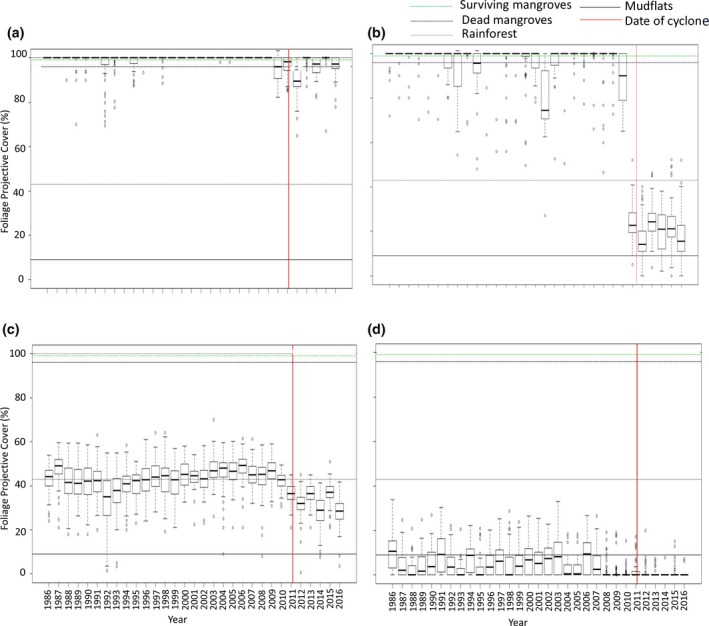
Change in foliage projective cover (FPC) (%) from 1986 to 2016 for (a) surviving mangroves, (b) dead mangroves, (c) other vegetation, and (d) mudflats. The horizontal lines indicate the respective mean values from 1986 to 2010 (prior to the cyclone) and the vertical line indicates Cyclone Yasi (March 3, 2011)

Prior to Cyclone Yasi, the FPC remained relatively stable for surviving mangroves with only a slight decrease noted following the cyclone (Figure 6a). A significant reduction in FPC to a level <30% (equivalent to nonvegetated regions, i.e., mudflats) was identified. In the 6 years following the cyclone, the FPC for the dead mangroves did not increase above 30% (Figure 6b). Prior to Cyclone Yasi, there were some years with a lower FPC than the mean, for both the surviving (Figure 6a) and dead mangroves (Figure 6b). This is likely due to disturbance events of lesser magnitude such as moderate flood events or lower category cyclones crossing the land further from Hinchinbrook Island. For instance, the reduced FPC in 1990 and 1991 is likely linked to a category 4 Tropical Cyclone Joy which crossed the coastline near Townsville (150 km south of Hinchinbrook Island). The other vegetation (i.e., rainforest) indicated seasonal FPC fluctuations with only a very slight reduction in FPC following cyclone Yasi (Figure 6c). Similarly, little variance in FPC was noted for the low intertidal mudflats with only a small reduction identified following the cyclone (Figure 6d). The classification of the four land cover classes used to generate the time series of FPC, HH, and HV backscatter was accurate to 92%.

### 5.3 Changes in L‐band HH and HV backscatter

**Figure 7:**
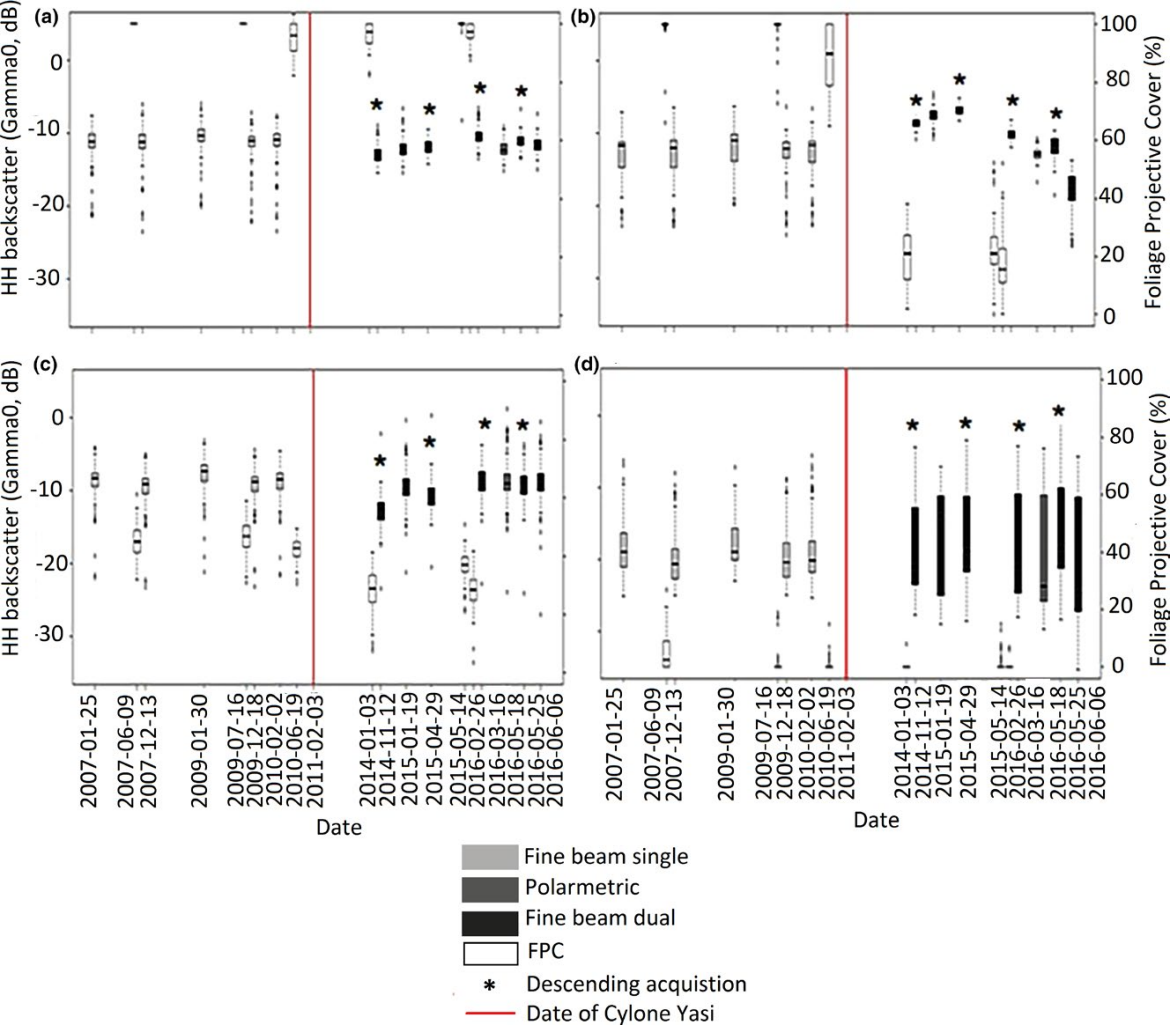
The change in the HH backscatter from ALOS‐PALSAR in relation to foliage projective cover (FPC) from 2007 to 2016 for: (a) surviving mangroves, (b) dead mangroves, (c) other vegetation, and (d) mudflats. The ALOS‐PALSAR images acquired with a descending angle of acquisition are identified by an asterisk; all other ALOS‐PALSAR images have an ascending angle of acquisition. The date of Cyclone Yasi is shown by the vertical gray line (February 3, 2011)

Prior to Cyclone Yasi (indicated by the red line in Figure 7), the HH backscatter was somewhat consistent across all land cover types, indicating the relative stability in the structural integrity of the landscape. As with the FPC, rainforest (Figure 7c) and mangroves unaffected by the cyclone (Figure 7a) exhibited a relatively constant HH (~−8 to ~−12 dB) backscatter following Cyclone Yasi (i.e., over the entire period of the time series). The exception was the mudflats for which the HH backscatter varied from ~−10 to ~−27 dB primarily because of the influence of tidal inundation (Figure 7d). Dead mangroves initially exhibited a higher response following the cyclone when compared to unaffected mangroves because of greater multiple scattering from woody debris (Figure 7b) with this then declining subsequently (by ~5–10 dB) as decomposition and removal of fallen wood occurred. The HV backscatter for surviving and dead mangroves increased from ~−20 to ~−10 (not shown), but then declined thereafter, with this also attributed in the presence of woody debris but exaggerated by above average rainfall, at the HV maximum (Table 1).

**Table 1:** The total rainfall compared to the mean rainfall for January and February measured at Cardwell Range (the nearest recording rainfall gauge to Hinchinbrook Island) (Bureau of Meteorology, 2016)

Year	Month	Total monthly rainfall (mm)	Mean monthly rainfall (1965 to 2016; mm)
2009	January	1267.4^a^	388.4
2009	February	1477.2^a^	540.7
2010	January	644.6^a^	388.4
2010	February	673.4^a^	540.7
2011	January	405.6^a^	388.4
2011	February	897.8^a^	540.7

a Indicates above average rainfall.

### 5.4 Area estimates of change

**Figure 8:**
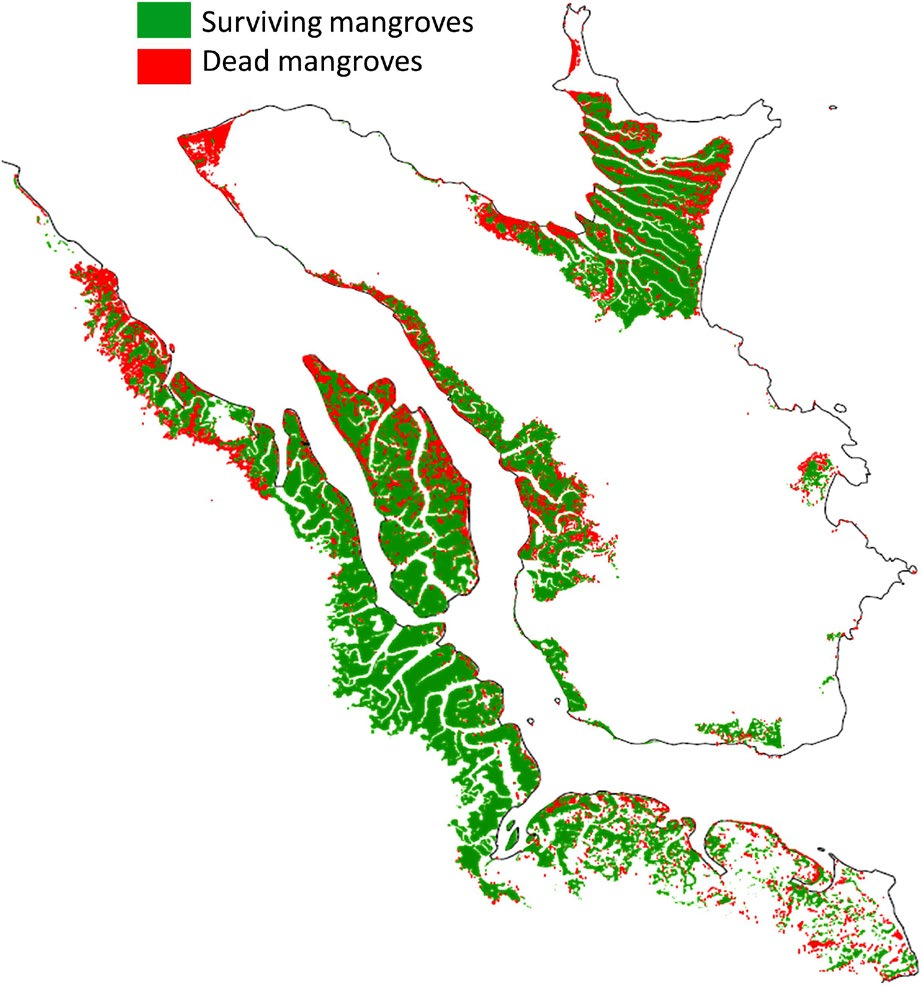
Distribution of clearings resulting from cyclone Yasi

**Figure 9:**
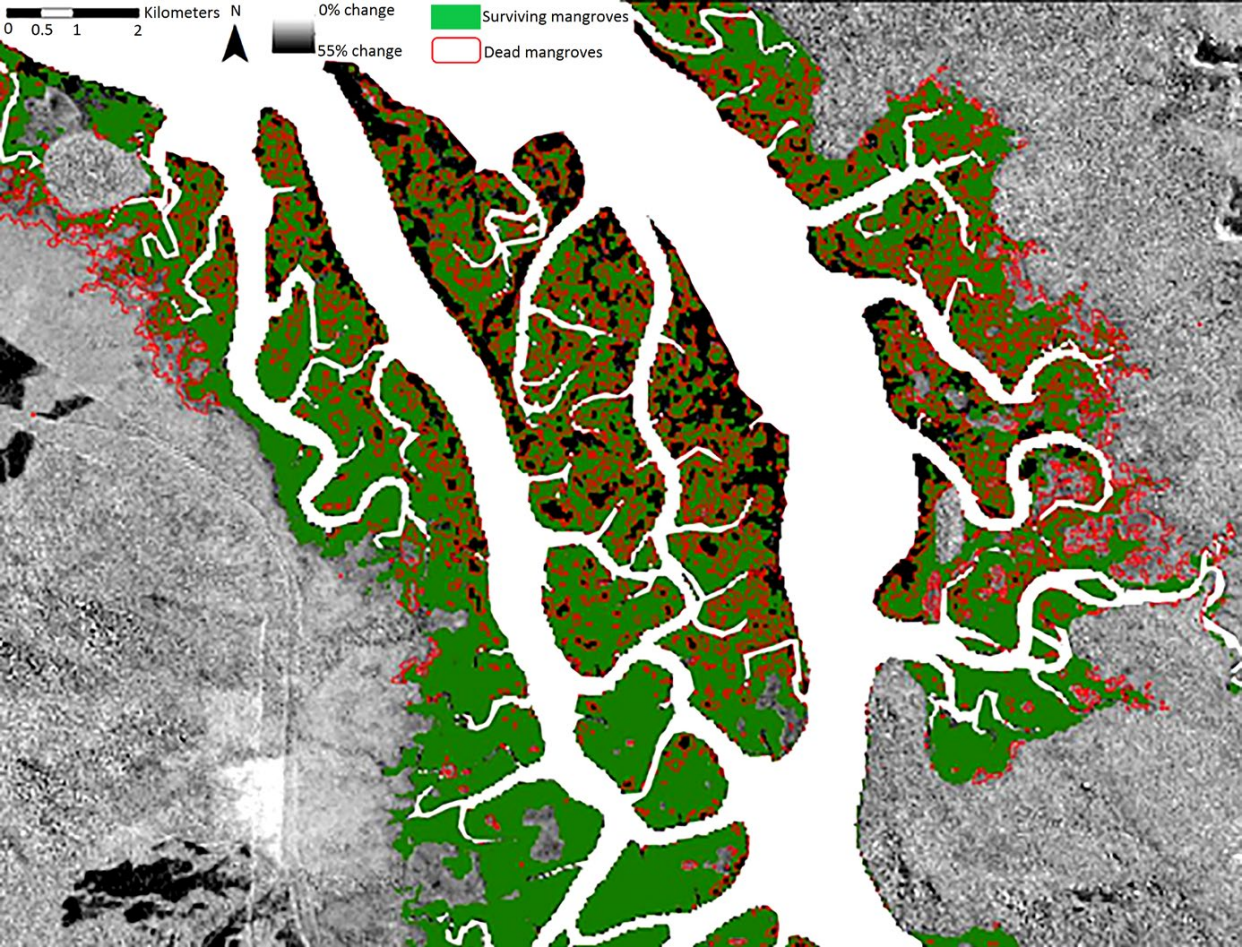
Percentage of FPC change between precyclone (2009) and postcyclone (2015) with defined regions of surviving and dead mangroves

The mangrove change map and subsequent areas of healthy and dead mangroves were identified and quantified to 81% accuracy. Following Cyclone Yasi (2015), 16% (2,178 ha) of mangroves were destroyed or degraded, with the majority of the damage occurring on the island within the Hinchinbrook Channel (26%, 461 ha) (Figure 8, Table 2). The national park zone is limited to Hinchinbrook Island (i.e., excludes the island within the channel and the Queensland coastline), and there was 20% (1,218 ha) loss of mangrove in this area. The majority of the damage was observed at the northwestern end of the island and at Missionary Bay. The Queensland coastline experienced minimal damage (8%, 499 ha). The FPC change prior to (2009) and following (2015) Cyclone Yasi indicated that the vast majority of the dead/damaged mangroves (shown in black) have not recovered 5 years following the cyclone (Figure 9).

**Table 2:** Area estimates prior to and following Cyclone Yasi

Region	Healthy mangroves prior to cyclone Yasi (2009) (ha)	Mangrove loss following cyclone Yasi (2015) (ha)	% Loss
Hinchinbrook Island National Park	5,989	1,218	20
Island within the Hinchinbrook channel	1,749	461	26
QLD coastline adjacent to Hinchinbrook Island	6,057	499	8
Hinchinbrook Island and the island within Hinchinbrook channel	7,738	1,680	21
All three regions combined	13,795	2,178	16

## 6 DISCUSSION

### 6.1 Temporal variations in foliage and wood material

During the period of observation prior to Cyclone Yasi (1986–2010), the Landsat FPC for nonmangrove vegetation (both rainforest and woodland combined) remained at approximately 40%, with little interannual variability. Similarly, the FPC of mangroves was >90%, which is typical of dense tropical vegetation given the dense foliage cover and limited seasonal variability in cover. Mudflats and other nonvegetated areas also exhibited very low FPC (<10%–20%), with a weak signal associated with sparse vegetation or mixed pixels. Defoliation and windthrow led to a loss of approximately 20%–30% FPC for nonmangrove vegetation and 10% for surviving mangroves. However, for mangrove communities, experiencing mortality, over 80% of the FPC was lost. The relatively high L‐band HH backscatter for nonmangrove forests (typically >10 dB) is typical of high biomass forests where double‐bounce interactions between the trunks, and large branches and the underlying surface dominate the signal (Lucas et al., 2010). The HH backscatter was approximately 2 dB lower for the surviving mangroves, which is attributable in part to their higher density and, for some species, disruption of double‐bounce scattering by the root systems (particularly prop roots; Lucas et al., 2007). The rapid increase in L‐band HH backscatter following Cyclone Yasi (to >−8 dB) was attributed to the increase in double‐bounce scattering between the now exposed ground surface, particularly if flooded, and fallen tree trunks and branches, which were directly observed in Queensland Globe imagery.

In subsequent years, the FPC of nonmangrove vegetation was variable but remained at the reduced levels observed following the cyclone with this attributed to the relatively slow regeneration of the open woodlands and forests which were effectively thinned during the event. By contrast, the FPC of surviving mangroves increased by about 5%–10%, with this suggesting that recovery of the canopy was in progress. However, the FPC of dead mangrove areas where windthrow had occurred remained at <80% throughout the postcyclone period indicating that minimal/no recovery had occurred. An increase in L‐band HH backscatter from affected areas was attributed to the presence of fallen trees, which were observed in the postcyclone Queensland Globe imagery. These increase the strength of the double‐bounce scattering (Lucas et al., 2010). However, decomposition and removal (e.g., during high tides) of the larger woody material leads to a subsequent decline to levels <−18 dB, which were typical of adjoining mudflats. No increase in L‐band HH backscatter was observed in subsequent years, suggesting that colonization and growth of mangroves were not occurring. In effect, the tree cover of these areas had been lost to mudflats following the cyclone with little sign of recovery. Variability in the SAR backscatter also occurred over time because of sensitivity to rainfall events, particularly at HV polarization.

### 6.2 Spatial variations in mangrove damage

The interpretation of the Queensland Globe aerial imagery indicated that many mangroves that had experienced windthrow were of darker coloration compared to those that were unaffected, with this being typical of those communities/zones dominated by *Rhizophora* species. Areas of lighter coloration were associated with mangroves species that were recorded in previous surveys (e.g., *Ceriops*,* Brugiuera,* and *Avicennia*). Therefore, the majority of the mangroves affected were dominated by tall and high biomass *Rhizophora* species. These stands of *Rhizophora* sp. were distributed throughout the mangrove zone, both on the fringes and internally, and hence, a scattered distribution of destruction was observed. The random pattern of spatial destruction may be due to the differential responses of the three Rhizophora species at Hinchinbrook Island (*R. lamarkii*,* R. stylosa,* and *R. apiculata*). Although there is a lack of studies comparing the interspecific differences in the response to cyclonic conditions, there is evidence in laboratory and field studies to suggest *R. apiculata* is less salt‐tolerant compared to *R. stylosa* (Ball et al., 1997) and *R. lamarkii* has the narrowest range of salinity tolerance preferring intermediate conditions, that is, mid‐shore (Duke, Ball, & Ellison, 1998). Depending on the distribution/zonation of these species, the tolerance to salinity changes during and following Cyclone Yasi may have contributed to the spatial pattern of destruction and recovery.

Based on this analysis of cyclone damage at Hinchinbrook Island, the mangroves had shown no sign of recovery after 6 years and this may be due to the physiology of *Rhizophora* propagules which prevents rapid recolonization of clearings in the interior of the forests. In some cases, the establishment of herbaceous species may also limit recovery as do changes in local conditions such as distance to mature trees, topography, inundation, and sediment chemistry that further limit mangrove recovery (Imbert, Bezard, Guitraud, Boraud, & Gross, 2000).

### 6.3 Why *Rhizophora* was affected over other species?

In other studies (Aung, Mochida, & Than, 2013; Roth, 1992; Smith Iii & Duke, 1987; Woodroffe & Grime, 1999), *Rhizophora* species have been shown to have a greater likelihood of mechanical damage compared to others. For example, following Cyclone Nargis in Myanmar in 2008, mangroves in the Rhizophoraceae group experienced 90% mortality, as opposed to non‐Rhizophoraceae with only 20% mortality (Aung et al., 2013). Bardsley (1985) investigated the response of 13 species of mangroves to Tropical Cyclone Kathy in the Gulf of Carpentaria in 1984, reporting that the majority of damage was due to *Rhizophora* species and attributed this to the high winds as opposed to tidal inundation. The greatest mortality was noted among the taller trees (which can exceed 15 m in northern Australia) as shorter communities were sheltered by the taller trees that were surrounding and by tidal inundation. However, persistent inundation was more likely to cause the loss of these smaller trees as a larger proportion of the plants would be submerged. Following Cyclone Tracy in 1974, Woodroffe and Grime (1999) studied the impact on mangroves in Shoal Bay (Beagle Gulf) in north Australia and reported that *R. stylosa*,* Ceriops tagal,* and *Bruguiera exaristata* had suffered the most damage, with this including windthrow, crown destruction, and bole fracture and reorientation. However, the majority of defoliated trees had remained relatively upright. Stoddart (1971) found that *Rhizophora* species suffered significant defoliation following Hurricane Hattie in Belize in 1961 but remained in an upright position.

There are several reasons why *Rhizophora* species are more susceptible to damage. First, the bole of the tree is supported by prop roots and as this is typically elevated above rather than being embedded in the underlying substrate, it is more vulnerable to snapping. The wood of *Rhizophora* species is also of a lower density and mechanical strength compared to other species such as *A. marina* (Santini, Schmitz, Bennion, & Lovelock, 2013). Wood density defines the resistance to high wind speeds such as those experienced during a cyclonic event (Niklas & Spatz, 2010; Saintilan, Rogers, Mazumder, & Woodroffe, 2013). For example, the wood of *Avicennia* species is comprised of alternating bands of xylem and phloem in a nonconcentric pattern which reduces the likelihood of radial splitting (from the center to the edge) and tangential splitting (from the top to the bottom) is more likely. This arrangement of vascular bundles ensures that, even when bole damage occurs, there are undamaged vascular bundles, thus allowing for the transport of water and nutrients to the crown, remaining branches, and epicormic shoots (Gill & Tomlinson, 1971; Saenger, 2002). This may therefore explain why stands of *Avicennia* species often remain relatively intact while *Rhizophora* species may experience damage to the boles and branches.

Finally, *Rhizophora* forests are among the tallest in world when mature compared to other species and are hence more exposed to strong winds, particularly high‐speed gusts. If the Rhizophora stands at Hinchinbrook Island were the tallest in 2011, this may explain the species‐specific mortality. Clough (1998) investigated the canopy structure at Hinchinbrook Island and found mixed stands of *B. gymhorrhiza* and *R. stylosa* at 14 m and shorter stands of *R. apiculata* and *R. stylosa* at 7 m. Age and subsequently height were identified as a controlling factor for destruction following Typhoon Haiyan in the Philippines. The tallest regions of Rhizophora (>46 years old) experienced approximately 100% mortality, whereas with younger, shorter trees completely recovered (Villamayor, Rollon, Samson, Albano, & Primavera, 2016).

*Rhizophora* species may also be vulnerable to inundation. For example, a storm surge in December 2004 impacted upon the Andaman and Nicobar Islands in the Indian Ocean resulting in mass mortality of *Rhizophora*‐dominated stands in South Andaman because of persistent inundation and sediment burial. The mangroves also experienced physiological stress in response to the increase in salinity as salt accumulation in leaves led to extensive defoliation. By contrast, *Avicennia* species were less affected, with this attributed to their greater tolerance to salinity (Cintrón, Lugo, Pool, & Morris, 1978; Ellison, 1993; Roy & Krishnan, 2005).

### 6.4 Why are the forests not recovering?

A major finding is that the mangroves that experienced the majority of damage during the cyclone are associated with *Rhizophor*a spp, have not recovered following the cyclone, and have been replaced by bare ground (unconsolidated sediments). The reasons for the lack of recovery relate primarily to the impact of high winds and rain during the cyclone and associated changes in hydrological and sedimentary regimes.

#### 6.4.1 Defoliation

The leaves of mangroves are essential for salt regulation, photosynthesis, and carbohydrate storage (Steinke & Ward, 1989; Tomlinson, 1986). Defoliation leads to a reduction in net productivity and greater resource allocation to the production of new leaves rather than the development of propagules (Anderson & Lee, 1995; Hodkinson & Hughes, 1982). While salt regulation and growth can occur with new leaves, reproductive success can be reduced considerably. Tong, Lee, and Morton (2003), in a defoliation experiment using *Kandelia candel* (Rhizophoraceae), identified that the number and size of propagules were significantly reduced up to a year after the defoliation event. Once defoliation occurs, mangroves tend to be more susceptible to environmental stressors including flooding, salinity, and disease (Anderson & Lee, 1995; Grace & Ford, 1996; Piyakarnchana, 1981). In the case of Hinchinbrook Island, many mangroves (particularly *Rhizophora*) were blown over and subsequently suffered mortality which was likely to have been hastened by the lack of foliage.

#### 6.4.2 Reductions in leaf sprouting capability

Another route to recovery is through sprouting of new leaves from reserve buds which, for *R. stylosa*, are located in the stems of younger trees but restricted to the terminal branches in more mature individuals (Bardsley, 1985). However, if trees lose their outer branches and hence the reserve buds as a consequence of wind damage, then recovery will be slow or not take place. As an illustration, Gill and Tomlinson (1977) established that *R. mangle* cannot regenerate branches larger than 2.5 cm in diameter. Following Cyclone Kathy in northern Australia in 1984, mature *Rhizophora* trees that had been defoliated showed limited recovery because of damage to the outer branches whereas *Avicennia* species rapidly recovered because of substantive growth of leaves (Bardsley, 1985). Snedaker, Brown, Lahmann, and Araujo (1992) also noted the lack of recovery of *R. mangle* following hurricane damage in Florida, whereas rapid recovery was observed for *Avicennia germinans* and *Laguncularia racemosa* partly due to sprouting from trees (primarily *A. germinans*) regardless of their orientation (upright or leaning) and the degree of leaf and branch defoliation. Similar observations were reported by Roth (1992, 1997), and Villamayor et al. (2016). Other studies have also reported limited recovery via coppicing and epicormic resprouting of mature *Rhizophora* sp. when branches containing the axillary and apical meristems are broken (Aung et al., 2013; Kauffman & Cole, 2010; Woodroffe & Grime, 1999). *Rhizophora* and *Ceriops* species also do not have reserve or secondary meristematic tissues and therefore are unable to recover through sprouting from the stump or root collar (Hamilton & Snedaker, 1984; Saenger, 2002). Age‐dependent mortality and recovery were also noted following Typhoon Haiyan in the Philippines in 2013 where mangroves older than 32 years experienced 95% mortality with no recover but those younger than 8 years fully recovered within 2 years despite mass defoliation (Villamayor et al., 2016). The viviparous nature of mangroves, particularly *Rhizophora* species, also allows them to colonize bare sediment and the aerial prop or stilt root system facilitates recovery from sedimentation as the lenticels are typically about 10 cm above the sediment surface. Hence, if the deposited sediment remains below the lenticels, the species will avoid the stress associated with burial (Ellison & Farnsworth, 1997; Paling et al., 2008).

### 6.5 Why is there no recolonization of cleared areas by other mangrove species?

The majority of mangroves that were damaged around Hinchinbrook Island had, in 2016, failed to recover, with this attributed to the high severity of the storm. When Typhoon Sudal made landfall in Micronesia in 2004, the majority of mangroves received direct wind damage and defoliation but the recovery was relatively rapid as approximately 80%–90% of trees remained standing and living (Kauffman & Cole, 2010). Hence, the damage was relatively temporary. However, if wind damage is more extensive and/or mangrove components (e.g., roots) are covered by sediment or inundated for long periods in the intertidal, recovery is slower particularly as the latter leads to adverse alterations to nutrients and gas exchange (Baldwin et al., 2001; Cahoon & Hensel, 2002; Smith et al., 1994; Smoak, Breithaupt, Smith, & Sanders, 2013). The recovery of the mangroves may also be limited by changes in substrate chemistry associated with the decomposition of fallen plant material (branches, roots, etc.). This decomposition results in anoxic sediments which decrease the redox potential and increase sulfide concentration, both of which limit propagule establishment (Cahoon et al., 2003; Mendelssohn et al., 1995). Reductions in live aerating root systems also reduce sediment oxygen balance and limit establishment of new propagules. Tidal inundation and changed sediment chemistry may explain the lack of recolonization following hurricanes in southern Florida, USA, despite the plentiful supply of propagules reaching the area due to dispersal along ocean currents (Swiadek, 1997). In the future, as sediment and inundation dynamics continue to change, the conditions may favor colonization of slower growing mangrove species such as *Ceriops*, whereas conditions may remain unsuitable for *Rhizophora*.

Significant erosion of the substrate during Cyclone Yasi due to strong winds and waves may have reduced the elevation below the threshold for successful propagule establishment, that is, permanent tidal inundation and high salinities. This was identified following Hurricane Mitch, which struck Mangrove Bight in Guanaja in Central America in 1998. The lack of propagule establishment and root growth resulted in no organic matter addition to the mangrove substrate, and decomposition of the remaining peat and soil organic matter led to sediment elevation collapse. Sediment collapse was predicted to continue for 10 years, assuming there was no organic sediment input or root growth (Cahoon et al., 2003). In these situations, dead mangrove forests may decompose and be replaced by mudflats, as was observed following Hurricane Donna in Florida in 1960 and is the potential reason for the lack of recovery at Hinchinbrook Island (Wanless, Tedesco, Risi, Bischof, & Gerlsanliter, 1995).

### 6.6 What are the implications for the long‐term future of mangroves?

Climate models and other predictions based on, for example, cyclonic histories and changes in atmospheric and ocean (sea surface) temperatures (Houghton et al., 2001; Hoyos, Agudelo, Webster, & Curry, 2006; IPCC, 2007) suggest (both globally and in Australia) an increasing although variable trend in the intensity of cyclones with an associated increase in mean and peak rainfall intensities, wind speeds, and wave heights. For instance, Walsh, Nguyen, and Mcgregor (2004) predicted a 56% increase in cyclone intensity by 2050, whereas Abbs (2012) predicts a 60% and 140% increase for 2030 and 2070, respectively. Furthermore, and particularly in the southern hemisphere, cyclone tracks may shift in a poleward direction (Abbs, 2012; IPCC, 2013, Leslie, Karoly, Leplastrier, & Buckley, 2007). However, there is still uncertainty and conflicts in predictions, partly because of the lack of complete cyclone records before 1970 (before regular satellite observations) (Abbs, 2012; Curry, Webster, & Holland, 2006). For example, within Australia, some studies (Leslie et al., 2007; Walsh et al., 2004) suggest the frequency will remain relatively constant while others (Abbs, 2012) suggest a decrease, particularly in Western Australia (44% decline by 2070). The duration of cyclones is also expected to decrease in the west and increase along the east coast (Abbs, 2012; Leslie et al., 2007).

In Australia, the paths, frequencies, and intensities of tropical cyclones are influenced by the ENSO patterns, with fewer cyclones occurring during El Niño periods (drier periods in south, east, and inland Australia) and more in La Niña years (above average rainfall in Australia, particularly in the north and central areas). Furthermore, the intensification of the Walker Circulation, which is typically characterized by strong easterly trade winds and westerly above grounds winds and often experienced during a La Niña, can result in multiple tropical cyclones within a relatively short period of time (Kuleshov, 2003; Kuleshov, Qi, Fawcett, & Jones, 2008; Nicholls, 1984). If cyclones increase in frequency and/or intensity, periods of inundation might increase which would potentially lead to reduced growth and establishment of propagules. However, extreme wind velocities may transport sediment and propagules to protected areas at higher elevations, thereby increasing local surface elevation. Species such as *Rhizophora* may become elusive in areas prone to cyclones and the changes in species composition and reductions in diversity may reduce the overall resilience of the forest. If a disturbance occurs that preferentially damages the remaining dominant species (e.g., *Avicennia*), then large sections of mangrove forest may experience mortality with this potentially resulting in the loss of the entire ecosystem.

## 7 CONCLUSION

The results from this investigation demonstrate that initial mangrove destruction following cyclonic activity is species specific with *Rhizophora* found to have the highest mortality due to wind damage, compared to surrounding stands most likely dominated by *Avicennia*. Following the cyclone, the regions of dead/damaged mangroves do not appear to increase, thereby suggesting little influence on long‐term secondary impacts such as changes in hydrological and sediment dynamics.

Seven years after cyclone Yasi, there was little recovery of *Rhizophora* species. This investigation presents a number of hypotheses for the species‐specific mortality and the lack of recovery. Although not yet verified through on the ground field data, the aim of identifying possible causes and potential reasons for change in accordance with similar postcyclone studies was achieved. The most likely cause for Rhizophora specific damage was attributed to the location of the reserve buds in the terminal branches of mature trees which are often damaged during cyclonic activity and also their inability to coppice or resprout. The mangrove clearings are not colonized by other mangroves species perhaps due to (a) significant changes in environmental conditions (i.e., hydrological regimes, sediment dynamics, sediment chemistry; anoxia, reduced redox potential, and increased sulfide concentrations), (b) dieback areas colonized by herbaceous terrestrial vegetation, perhaps saltmarsh species, and (c) the high proportion of mortality in the inner forest where there is a lack of nutrients and inundation stress.

The sensitivity of *Rhizophora* to mechanical damage and lack of rapid efficient recovery may explain the absence of tall mature *Rhizophora* stands in cyclone prone regions such as the Sundarbans, which is impacted by 30–40 typhoons each year in the adjacent Bay of Bengal (Smith Iii & Duke, 1987; Villamayor et al., 2016). By contrast, 40–45 m *Rhizophora* forests thrive in sheltered cyclone‐free locations such as Ecuador, South America (FAO, 2007).

Climate change trajectories suggest a decrease in the number of tropical cyclones, as systems occur when there is a strong vertical temperature gradient between the surface of the Earth and the upper atmosphere. As the atmospheric temperature is predicted to increase the vertical temperature gradient will weaken. However, cyclone intensity is predicted to increase as the warmer sea surface temperatures present a larger source of energy (heat) for the cyclones to draw energy from (Emanuel, 2000; IPCC, 2013; Wing, Sobel, & Camargo, 2007). The impact on mangrove ecosystems will vary depending on site‐specific characteristics such as geomorphology, climate, species composition, sediment availability, and hydrological regimes. Forests with low diversity and dominated by vulnerable species, in cyclone prone regions, will be vulnerable to mass mortality. Forests at risk should be identified and management strategies implemented to prevent the loss of entire ecosystems and the secondary negative implications for the local economy and adjacent ecosystems (i.e., seagrasses and coral reefs).

## AUTHOR CONTRIBUTION

E.A, R.L, and A.A conceived the presented idea. E.A performed the data analysis, and R.L and K.R helped with technical difficulties. E.A wrote the manuscript, with input and edits from R.L, A.A, and K.R.

## 8 DATA ACCESSIBILITY

All mapping for Hinchinbrook Island prior to, and following Cyclone Yasi, is available online on the Terrestrial Ecosystem Research Network (TERN) together with metadata sheets. http://www.tern.org.au/.
